# Treatment satisfaction and medication adherence and predictors among patients with heart failure in Ethiopia: a hospital-based cross-sectional study

**DOI:** 10.3389/fphar.2024.1399177

**Published:** 2024-07-29

**Authors:** Abate Wondesen Tsige, Bedilu Linger Endalifer, Habtemariam Alekaw Habteweld, Yehualashet Teshome Wondmkun, Siraye Genzeb Ayele, Belayneh Kefale

**Affiliations:** ^1^ Department of Pharmacy, College of Health Sciences, Debre Berhan University, Debre Berhan, Ethiopia; ^2^ Department of Midwifery, School of Nursing and Midwifery, College of Health Sciences, Addis Ababa University, Addis Ababa, Ethiopia; ^3^ Clinical Pharmacy Unit and Research Team, Department of Pharmacy, College of Medicine and Health Sciences, Bahir Dar University, Bahir Dar, Ethiopia

**Keywords:** treatment satisfaction, medication adherence, heart failure, Ethiopia, Debre Berhan comprehensive specialized hospital

## Abstract

**Background:**

Globally, about 18 million people died from cardiovascular diseases (CVDs) in 2019, over three-quarters in developing countries. Non-adherence to medication in CVD patients causes hospitalization, worsened symptoms, higher healthcare costs, and more emergency visits. Hence, this study aimed to assess treatment satisfaction and medication adherence and predictors in heart failure (HF) patients attending Debre Berhan Comprehensive Specialized Hospital (DBCSH), Ethiopia.

**Methods:**

A hospital-based cross-sectional study was undertaken at the medical referral clinic of DBCSH. A total of 344 ambulatory HF patients who visited the medical care of the DBCSH medical referral clinic during the study period were included. Treatment satisfaction was assessed using a self-administered Medicine Questionnaire (SATMED-Q). Relationships between predictor variables and treatment satisfaction were determined using one-way analysis of variance (ANOVA) and an independent *t*-test. Medication adherence was determined using the Morisky Green Levin Medication Adherence Scale (MGLS).

**Results:**

Participants with drug-drug interactions (DDIs) were approximately 38% less likely to adhere to medication compared to their counterparts (AOR = 0.62, 95% CI: 0.54–0.71). Additionally, participants who had taken five or more drugs were approximately 68% less likely to adhere to medication compared to those who had taken only one drug (AOR = 0.32, 95% CI: 0.2–0.51). The correlation between medication adherence and drug-drug interactions remains a possible pseudo-correlation via the number of medications taken. There was a noteworthy positive correlation (rs = 0.34, *p* = 0.027) between participants’ treatment adherence and treatment satisfaction.

**Conclusion:**

The rate of treatment satisfaction and treatment adherence among HF patients was 67.6% and 60.9%, respectively. The presence of DDI and the number of drugs were identified as predictors to medication adherence.

## Introduction

Cardiovascular diseases (CVDs) cause 17.9 million deaths occurred in developing nations (WHO, 2021). In Sub-Saharan African (SSA) countries, poor access to high-quality and inaccessible healthcare contributes to an increase in CVD morbidity and death (Mensah et al., 2015; Meyer et al., 2017; Schultz et al., 2018; Rosengren et al., 2019). The prevalence of CVD in Ethiopia was 5,534 per 100,000 people (Ali et al., 2020).

Globally, heart failure (HF) constitutes a significant medical and economic challenge (Lesyuk et al., 2018). It results from changes in cardiac structure or function that impair the ability of the ventricle to fill with or eject blood (Parker et al., 2020). The incidence and prevalence of HF are increasing; approximately 6.5 million Americans currently have HF, with 1,000,000 new cases diagnosed each year and annual expenditures exceeding 30 billion US dollars (Benjamin et al., 2018). Based on the phenotypes of the disease, HF can be classified as HF with reduced ejection fraction (EF) (HFrEF) and HF with preserved EF (HFpEF) (Mann et al., 2018).

Managing HF remains challenging due to co-existing co-morbidities (Chiatti et al., 2012). In addition, prescribing a higher number of medications for HF patients results in non-adherence to medication and more frequent hospital stays (Page et al., 2016). To achieve favorable clinical outcomes, HF guidelines emphasize the paramount importance of adhering to prescribed treatment regimens (Riegel et al., 2011; Ponikowski et al., 2016). Maximizing treatment results depends critically on adherence to HF medications (Pallangyo et al., 2020).

Studies conducted in various countries indicated that HF patients adhering to their medication experienced fewer emergency department visits, improved clinical survival, fewer HF exacerbations, and lower healthcare costs (Hope et al., 2004; Murray et al., 2009; Wu et al., 2009). However, medication non-adherence was associated with increased hospitalization (Knafl and Riegel, 2014a; Aggarwal et al., 2015), worsening symptoms, disease progression, an overall increase in healthcare costs (Aggarwal et al., 2015), and frequent emergency department visits (Davis et al., 2014). Heart failure medication adherence often falls below optimal levels in global investigations using various adherence evaluation methods (Fitzgerald et al., 2011; Lee et al., 2015; Shah et al., 2015; Amininasab et al., 2018; Fernandez-Lazaro et al., 2019; Rehman et al., 2019; Pallangyo et al., 2020).

Patient satisfaction with prescriptions or services influences treatment outcomes, the duration of pharmacological care, optimal service utilization, clinical compliance, and treatment plan adherence (Langebeek et al., 2014).

Patients satisfied with their treatment adhere more to prescribed therapeutic regimens, take an active role in their self-care, and improve their quality of life compared to those dissatisfied with their therapy (Asadi-Lari et al., 2003; Liberato et al., 2016). Individuals treated satisfactorily with services are more likely to remain members of the healthcare facility and adhere to prescribed medication regimens (Holsclaw et al., 2005). Medication non-adherence in HF patients ranged from 18% (Simpson et al., 2021) to 92% (Chang et al., 2018). While the rate of treatment satisfaction among HF patients was 4.2 mean score out of 5 best scores (Bjertnaes et al., 2012). However, no study has assessed treatment adherence and patient satisfaction among HF patients at DBCSH, Ethiopia. Hence, this study aims to evaluate treatment satisfaction and medication adherence and their predictors, which influence treatment outcomes in HF care.

## Material and methods

### Study setting and participants

A cross-sectional study was conducted on ambulatory HF patients who visited the DBCSH medical referral clinic for HF care from 30 January 2021, to 30 April 2021. The medical referral clinic, one of the specialty clinics in DBCSH, provides cardiac care.

### Eligibility criteria

#### Inclusion criteria

Patients with HF receiving follow-up at an adult ambulatory medical referral clinic of DBCSH, aged 18 years or older, and those with complete medical records were included in the current study.

#### Exclusion criteria

Study participants who refuses to give informed consent, too sick patient during the interview, admitted patients and those missing their appointment data were excluded from the current study.

### Sample size determination and sampling technique

The sample size was estimated using a single population proportion formula. Taking treatment satisfaction proportion in HF patients was 50% to get the possible minimum sample size.

$$n=\frac{\;Z{\;\frac{\alpha }{2}}^{2}\;p\left(1‐p\right)}{d^{2}}\;$$

Where n - is the minimum sample size required for a large population (≥10,000)

Z α/2 - is the critical value for a 95% confidence interval (1.96 from Z-table)

p - Prevalence = 50% (0.5)

d - Degree of accuracy desired (the margin of error 5% = 0.05); then the sample size is

$$n=\frac{\;{\;\left(1.96\right)}^{2}\times \left(1‐0.5\right)}{{\left(0.05\right)}^{2}}=384.18=∼384$$

The expected number of source population in the study period (N), based on the average number of patients coming to the clinic 3 days a week with a total of 16 weeks was 1,680 (16 × 3 × 35). The corrected sample size, using the following correction formula was 312.6 ≈ 313, Corrected sample size = $\frac{\;n\times N}{n+N}$

Then 10% contingency was added on 313: 313% × 10% = 32.

The final sample size included in the current study was 344.

A systematic random sampling technique was used to select study participants from the Health Management Information Systems (HMIS) list of HF patients at the medical referral clinic of DBCSH.

### Data collection procedures and tools

Two nurses and one clinical pharmacist underwent training for data collection, with pharmacists handling clinical data review and nurses conducting patient interviews.

Clinical and demographic data of study participants were collected using pre-tested data abstraction tools and structured questionnaires.

Drug-drug interactions were assessed using Micromedex drug interaction checkers, drugs.com, and up-to-date.

Treatment satisfaction was assessed using the self-administered Medicines Questionnaire (SATMED-Q), consisting of 17 items across six domains: treatment effectiveness (3 items), undesirable side effects (3 items), impact on daily activity (3 items), medical care (2 items), convenience of use (3 items), and global satisfaction (3 items). Each item in a specific domain received an ordinal score on a five-point Likert scale: not at all (0), a little bit (WHO, 2021) point, somewhat satisfied (Schultz et al., 2018) points, quite a bit (Mensah et al., 2015) points, and very much satisfied (Rosengren et al., 2019) points. The sum of the items ranged from 0 to 68 points, with higher scores indicating greater patient treatment satisfaction with the drug therapy. This score was transformed into a more intuitive and easily understandable metric, ranging from a minimum of 0 to a maximum of 100, using the following formula:

$$Y’=\left[\left(\text{ Yobs}‐\text{Ymin}\right)\;/\;\left(\text{Ymax}‐\text{Ymin}\right)\;\right]\times 100=\text{Yobs}\times 1.471$$

Where Ymax is 68 (maximum total score); Ymin is 0 (minimum total score); Yobs is the total score obtained by the patient; and Y′ is the transformed score. A similar expression can be used to change the metric of each domain (Ruiz et al., 2008; Rejas et al., 2011).

Medication adherence was determined using the Morisky Green Levin Medication Adherence Scale (MGLS). It has four items focusing on past medication use patterns with closed dichotomies (yes/no). Each ‘yes’ response was rated as 0 and each ‘no’ response was rated as 1. The total summed score ranges from 0 to 4 and was grouped as good adherence to medication (0–2 points scored) and non-adherence to medication (≥3 points scored) (Beyhaghi et al., 2016).

### Data analysis and interpretations

Data entry was conducted using Epidata version 4.2.0, while data analysis was performed using Statistical Package for Social Sciences (SPSS) version 25 software. Descriptive statistics, including frequency, mean, and percentage, were employed to summarize study participant characteristics.

The relationships between predictor variables and treatment satisfaction were assessed using one-way analysis of variance (ANOVA) with *post hoc* analysis for mean values of more than two continuous variables. For the mean values of two continuous variables, an independent *t*-test was employed. Binary logistic regression analysis was used to examine the association between predictor variables and medication adherence.

The relationship between the treatment adherence score and total treatment satisfaction scores was elucidated using Spearman’s correlation coefficient.

### Ethics approval

The study (P009/01/2021) received ethical clearance from the Debre Berhan University Institutional Review Board. Written informed consent was obtained from all study participants who accepted the invitation to participate. All methods were conducting in accordance with the relevant guidelines and regulations (we followed the declaration of Helenski).

## Results

### Clinical characteristics and demographic features

Study participants had a mean age of 53.38 (SD, 18.84) years, and most (45%) were in the age range of 31–60 years. Most of them were females (65.1%), married (45.1%), and residents of rural areas (54.4%). A drug allergy history was not found in 98.6% of study participants. Most study participants (72.7%) had less than 4 years of follow-up with DBCSH (Table 1).

**TABLE 1:** Socio-demographic and clinical characteristics of study participants at DBCSH.

Variables	Categories	Number (%)
Gender	Male	120 (34.9)
	Female	224 (65.1)
Age in years	18–30	56 (16.3)
	31–60	155 (45)
	>60	133 (38.7)
Relational status	Single	78 (22.7)
	Married	155 (45.1)
	Widowed	64 (18.6)
	Divorced	47 (13.7)
Educational attainment	Unable to write or read	120 (34.9)
	Primary	71 (20.6)
	Secondary	74 (21.5)
	A diploma or higher	79 (23.0)
Location of residence	Urban	157 (45.6)
	Rural	187 (54.4)
Occupation	Governmental	57 (16.6)
	Working at private company	84 (24.4)
	Unemployed	99 (28.8)
	Housewife	25 (7.3)
	Merchant	44 (12.7)
	Retired	14 (4.1)
	Others^a^	21 (6.1)
The source of the drugs
	Hospital covered	176 (51.2)
	Self-care	147 (42.7)
	Insurance coverage	21 (6.1)
Subsequent years	≤4 years	250 (72.7)
	≥5 years	94 (27.3)
How often do you follow	≤3 months	341 (99.1)
	≥3 months	3 (0.9)
Drug allergy background	No	339 (98.6)
	Yes	5 (1.4)

^a^ Guard, Mechanics, and Student.

### Treatment satisfaction of HF patients

Considering that the scores of the SATMED-Q ranged from 0 to 100 in each domain, the higher the score, the greater the treatment satisfaction with the medicine. The overall treatment satisfaction score of the study participant was 67.6(SD, 11.33). The highest scores were found in the treatment effectiveness 83.08 (SD, 2.04) domain, and the lower scores were reported in the impact on daily activities 80.42 (SD, 2.14) and global satisfaction 72.58 (SD, 2.10) domains. The lowest scores were reported in the side effects 10.33 (SD, 1.62) domain (Table 2).

**TABLE 2:** Treatment satisfaction in HF patients at the medical referral clinic of DBCSH.

Domain items		Min	Max	Percentage	Mean	SD
Undesirable side effects domain		0	12	10.33	1.24	1.62
1	The side effects of the medicine interfere with my physical activities	0	14	4	0.56	0.51
2	The side effects of the medicine interfere with my Leisure and free time activities	0	13	4	0.52	0.61
3	The side effects of the medicine interfere with my daily activities	0	4	4	0.16	0.50
Treatment effectiveness domain		0	12	83.08	9.97	2.04
4	The medicine I am taking relieves my symptoms	0	81.25	4	3.25	0.73
5	I am satisfied with the time it takes for the medicine to start to work	0	87.5	4	3.50	0.67
6	I feel better now than I did before starting the treatment	0	80.5	4	3.22	0.64
The convenience of using the domain		3	12	82.42	9.89	2
7	I find that taking my medicine is practical for me	1	84.75	4	3.39	0.63
8	I find it easy to use/take the medicine in its present form (taste, size, etc.)	1	80.25	4	3.21	0.66
9	The timetable for taking the medicine suits me	1	82.25	4	3.29	0.71
Impact on daily activities domain		1	12	80.42	9.65	2.14
10	Thanks to the medicine I am taking, it is easier for me to do my leisure and free time activities	0	80.5	4	3.22	0.71
11	Thanks to my medicine, it is easier for me to take care of my hygiene	1	80.5	4	3.22	0.72
12	Thanks to my medicine, it is easier for me to perform my daily activities	0	80.5	4	3.21	0.71
Medical care domain		2	8	81	6.48	1.43
13	My doctor has informed me in detail about my medical condition	0	80.75	4	3.23	0.82
14	My doctor has informed me about the right way to treat my medical condition	2	81.25	4	3.25	0.61
Global satisfaction domain		2	12	72.58	8.71	2.10
15	I intend to continue using this treatment	2	76.25	4	3.05	0.73
16	I feel comfortable with my treatment	0	71.5	4	2.86	0.70
17	In general, I feel satisfied with the treatment	0	70	4	2.80	0.67
Total score		8	68	67.6	45.94	11.33

Min: Minimum; Max: Maximum; SD: standard deviation.

### Treatment satisfaction of HF patients’ relationship with different characteristics of study participants

Participants with a diploma and above education level (mean = 68.73, SD = 5.82), those with comorbidities (mean = 67.82, SD = 6.12), those took one drug, individuals not adhering to salt restrictions (mean = 68.45, SD = 6.52), and participants who had no drug-drug interaction (mean = 68.1, SD = 5.23) exhibited notably higher treatment satisfaction scores than their counterparts (*p* < 0.05). No significant relationship was observed between treatment satisfaction and the other reported demographic and clinical characteristics (*p* > 0.05) (Table 3).

**TABLE 3:** Relationship between treatment satisfaction and different demographic and clinical characteristics of patients with HF.

Variables	Category	N	Mean SATMED-Q score ± SD	F	*p*-value
Co-morbidity	No	148	66.2 ± 6.36		
	Yes	196	67.82 ± 6.12	1.03	0.02*
Sex	Male	120	66.69 ± 6.04		
	Female	224	67.36 ± 6.39	0.18	0.33*
Educational level	No formal education	120	66.70 ± 5.91		
	Primary	71	65.33 ± 8.21		
	Secondary	74	67.85 ± 4.52		
	Diploma and above	79	68.73 ± 5.82	4.31	0.005**
Age	18–38	82	66.14 ± 6.71	2.61	0.075**
	39–59	124	68.08 ± 6.51		
	≥60	138	66.88 ± 5.68		
Number of Comorbidity	None	121	67 ± 6.62	0.03	0.9**
	1	156	67.19 ± 5.49		
	≥2	67	67.18 ± 7.31		
Source of medications	Free	176	67.59 ± 6.78	1.91	0.15**
	Paid	147	66.4 ± 5.73		
	Covered by insurance	21	68.37 ± 4.98		
Area of residence	Rural	157	66.89 ± 5.97	0.49	0.51*
	Urban	187	67.33 ± 6.52		
Drug allergy history	No	337	67.12 ± 6.27	0.000	0.82*
	Yes	7	67.67 ± 6.52		
Salt restriction	No	69	68.45 ± 6.52	0.11	0.049*
	Yes	275	66.79 ± 6.17		
Drug-drug interaction	No	167	68.1 ± 5.23	0.62	0.038*
	Yes	177	66.23 ± 6.14		
Number of drugs	One	27	69 ± 6.71	0.14	0.03**
	2–4 drugs	171	67.8 ± 5.68		
	≥5 drugs	146	66 ± 5.90		

SATMED-Q; Satisfaction with Medicines Questionnaire: SD; standard deviation.

*Independent *t*-test.

**One-way analysis of variance (ANOVA).

Bold values; indicate variables which had significant relationship with treatment satisfaction.

### Rates of study participants’ medication adherence

**FIGURE 1:**
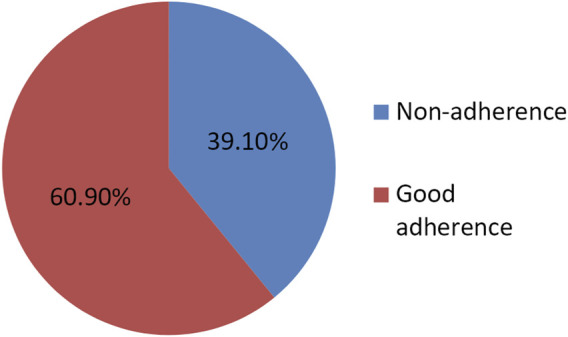
Rates of study participants’ medication adherence.

Nearly two-thirds of the study participants exhibited good treatment adherence, with 217 individuals (60.9%), while 127 participants (39.1%) showed low treatment adherence levels (Figure 1).

### Contributing factors for medication adherence

In the binary logistic regression analysis, the number of prescribed drugs and occurrences of DDIs were significantly associated with treatment adherence. Study participants who had drug-drug interactions were about 38% less likely to be on medication adherence than participants who did not have drug-drug interactions (AOR = 0.62, 95% CI: 0.54–0.71). In addition, study participants who had taken more than or equal to five drugs were about 68% less likely to adhere to medication than study participants who had taken one drug (AOR = 0.32, 95% CI: 0.2–0.51) (Table 4). The number of medications and drug-drug interactions correlate with medication adherence, respectively, but did not have a causal relationship.

**TABLE 4:** Univariable and multivariable analysis of factors associated with treatment adherence in heart failure patients.

Variables	Categories	Adherence		OR (95% CI)	
		Non-adherent	Adherent	COR	AOR
Sex	Male	80	40	1	1
	Female	156	68	0.85 (0.55–1.33)	1.12 (0.65–1.91)
Age (years)	18–30	69	67	1	1
	31–60	61	42	1.11 (0.59–2.07)	1.25 (0.51–3.07)
	>60	104	69	0.63 (0.36–1.11)	1.99 (0.79–5.02)
Occupation	Governmental Employed	41	57	1	1
	Private employed	39	46	0.70 (0.38–1.30)	1.87 (0.69–5.12)
	Unemployed	34	44	0.69 (0.37–1.29)	1.22 (0.45–3.31)
	Self-employed	22	27	0.95 (0.45–2.03)	1.25 (0.45–3.50)
	Others*	17	17	0.49 (0.22–1.13)	1.44 (0.46–4.49)
Adverse drug reaction	No	300	39	1	1
	yes	2	3	1.16 (0.36–3.78)	1.31 (0.33–5.26)
Drug-drug interaction	No	100	67	1	1
	Yes	106	71	0.43 (0.27–0.68)	**0.62(0.54–0.71)**
Source of medications	Free	40	30	1	1
	Paid	100	79	0.96 (0.53–1.74)	0.72 (0.35–1.48)
	Covered by insurance	24	20	0.50 (0.23–1.07)	0.41 (0.16–1.01)
	Covered by family	32	19	0.61 (0.28–1.34)	0.48 (0.19–1.22)
Presence of co-morbidity	No	55	44	1	1
	Yes	151	94	0.50 (0.29–0.85)	1.58 (0.84–2.95)
Number of drugs	One	14	13	1	1
	2–4 drugs	100	71	0.95 (0.27–3.39)	0.97 (0.84–12.9)
	≥5 drugs	80	66	0.19 (0.05–0.65)	**0.32(0.2–0.51)**

OR; odd ratio, COR; crude odd ratio, AOR; adjusted odd ratio, CI; confidence interval.

Bold values; indicate variables which had significant association with treatment adherence.

### The relationship between treatment satisfaction and treatment adherence

There was a significant positive correlation between treatment satisfaction and medication adherence (rs (342) = 0.34, *p* = 0.027).

## Discussion

The study revealed that a treatment satisfaction rate of 67.6% and a medication adherence rate of 60.9% among HF patients.

In medical studies, knowing how satisfied patients are with their treatment is essential to understanding their perspective on care. It is also shown that improving clinical outcomes is connected to increasing patient satisfaction with their care (Al-Jabi et al., 2015).

In this study, most participants were aged 30–59, with an average age of 53.4 years. This was lower than a study in Brazil (average age 60.2, range 28–87) (), but similar to a study in Nigeria (average age 52, range 32–83) (Iloh and Amadi, 2017). But, the study conducted in Greek reported that 74% of participants found in the age range of 18–59 years (Geitona et al., 2008). Age among HF patients was linked to both treatment satisfaction and medication adherence.

More than two-thirds of participants had a co-morbid illness, a finding similar to a Palestinian study (63.2%) (Al-Jabi et al., 2015). Almost three-fourths of the study subjects had received treatment for 4 years or less, in contrast to research in Palestine (Al-Jabi et al., 2015), where the majority had been treated for more than 4 years (69.7%). The variations could be differences in the study participants’ clinical characteristics.

In the current study, participants had an overall treatment satisfaction score was 67.6%. This finding was similar with a study done in Brazil (69.2%) (). However, this result was lower to studies conducted among cancer patients in Greece (85.6%) (Pini et al., 2014), Tikur Anbessa Specialized Hospital, Addis Ababa, Ethiopia (80.81) (Demoz et al., 2019), Nigeria (78.6%) (Iloh and Amadi, 2017), Greek (more than 80%) (Geitona et al., 2008) but higher than studies from Australia (nearly 65% fully satisfied only) (Candlish et al., 1998) and Addis Ababa, Ethiopia (Seid et al., 2020). These differences may be variations in the definition of treatment satisfaction between studies. Patient satisfaction with therapy is the most reliable indicator of continued medication use, impacting the effectiveness and efficiency of medical care (Zyoud et al., 2013; Iloh and Amadi, 2017). So, satisfaction with medication constitutes a quality indicator that can be used for improving healthcare of chronic patients like HF. The findings imply that barriers to treatment satisfaction of HF patients must be addressed.

The satisfaction scores of the study participants regarding medication side effects were relatively low (10.33%) compared to other domains. This result contrasts with a study in Addis Ababa, Ethiopia (79.3%) (Seid et al., 2020), a study in Palestine (86.0%) (Hope et al., 2004), Brazil (93.5%) (), . The discrepancy may be attributed to differences in the study setting, the number of comorbidities the study participant has, number and types of medication study participants take, severity of illness or the side effect items are being measured incorrectly. The Addis Ababa study by Seid et al. was conducted at a tertiary hospital, where patients might have multiple comorbidities and take multiple drugs, potentially leading to more drug side effects among participants. In addition, it is possible that the patients who participated in this study have different understanding of side effects, different perception of side effects, different perception of the severity of side effects, and different interpretation of the SATMED-Q questions.

The global treatment satisfaction domain score was 72.6% in the current study compared to other dimensions. This result was consistent with a study conducted in Palestine (72.1%) (Al-Jabi et al., 2015), but this finding was greater than a study done in Brazil (69.2%) (). The score in the medical care domain was 81%. This finding was higher than a study done in Estonia (68%) (Polluste et al., 2000). A possible reason for this variation could be the treatment satisfaction assessment tools used.

The occurrence of comorbidity, individuals with a diploma or higher educational level, and those with no salt reduction showed a statistically significant relationship with treatment satisfaction. However, other clinical and socio-demographic characteristics did not exhibit a statistically significant relationship with treatment satisfaction. Additionally, our result was inconsistent with studies reported from China (Fang et al., 2019) and Saudi Arabia (Ammo et al., 2014). Primary factors associated with low satisfaction with healthcare services include waiting time, extensive administrative procedures, appointments, and the attitudes of medical personnel toward patients (Zyoud et al., 2013; Lazarevik and Kasapinov, 2015). Our finding was contradicted with a web-based survey conducted in Macedonia, Serbia, and Bulgaria (Lazarevik and Kasapinov, 2015).

In the current study, most heart failure (HF) patients showed good treatment adherence (60.9%). This result was higher than a study in Yemen (45.8%) (Alakhali et al., 2013) but lower than in the Brazil (77%) (Da Silva et al., 2015).

It also aligned with findings from other studies (Murray et al., 2007; Bisharat et al., 2012; Zhao et al., 2015). These variations could be due to differences in how treatment adherence is measured, the patient-care strategies used by pharmacists, and differences in the definition of adherence. For example, our study was conducted at DBCSH, a referral hospital in a resource-limited setting, with participants dealing with complex medical conditions and multiple medications, leading to a lower treatment adherence rate compared to other study locations. Low adherence among patients with HF is adversely affecting clinical results and leading to greater HF exacerbations, lower physical activity, and a greater likelihood of hospitalization and mortality. Our study found that study participants had a low medication adherence rate; thus, effective interventions are needed to increase medication adherence and achieve improved medical outcomes.

Participants taking five or more drugs were 68% less likely to adhere to treatment compared to those using only one drug. This finding aligns with studies conducted in the United States of America and Iran, emphasizing that a higher pill burden could decrease treatment adherence (Knafl and Riegel, 2014b).

### Strengths and limitations of the study

This study has several limitations and strengths. The cross-sectional nature of the study precludes establishing a causal relationship between the demographic and clinical characteristics of study participants and the outcome variable. However, there is possibility of pseudo-correlation remains for drug-drug interactions, and it is not clear whether there is a causal relationship or not. The collected data was primarily obtained from study participants’ self-reports during interviews. Skipping essential information and recall bias may impact the study’s overall treatment satisfaction and adherence. Despite these limitations, this study suggests that healthcare personnel should prioritize counseling HF patients about symptoms, and non-pharmacological and pharmacological treatment to enhance treatment satisfaction and medication adherence.

## Conclusion

In conclusion, the study revealed a treatment satisfaction rate of 67.6% and a medication adherence rate of 60.9% among HF patients. The statistically significant association between drug-drug interactions (DDI) and the number of drugs used highlights the importance of addressing these factors for improving treatment adherence. The correlation between medication adherence and drug-drug interactions remains a possible pseudo-correlation via the number of medications taken. Therefore, it is imperative for medical facilities to ensure the provision of quality services and all necessary resources to enhance treatment satisfaction and medication adherence among heart failure patients. This may involve optimizing healthcare processes, reducing drug interactions, and promoting comprehensive patient care.

## Data Availability

The raw data supporting the conclusion of this article will be made available by the authors, without undue reservation.
